# Editorial: Autism Signaling Pathways

**DOI:** 10.3389/fncel.2021.760994

**Published:** 2021-09-28

**Authors:** Yi-Ping Hsueh, Yu-Chih Lin

**Affiliations:** ^1^Institute of Molecular Biology, Academia Sinica, Taipei City, Taiwan; ^2^Program in Neuroscience, Hussman Institute for Autism, Baltimore, MD, United States

**Keywords:** autism spectrum disorders, cell adhesion, G-protein coupled receptor, IL-17A, parvalbumin, repetitive behaviors, transcriptomic analysis, valproic acid

## Introduction

Autism spectrum disorders (ASD), a group of heterogeneous neurodevelopmental disorders, are characterized by two main behavioral features, termed the ASD dyad (https://www.cdc.gov/ncbddd/autism/hcp-dsm.html). The first is defective social interaction and impaired verbal and non-verbal communication. The second is abnormal sensory responses and stereotypic repetitive behaviors (Grzadzinski et al., 2013). As a neurodevelopmental disorder, genetic variation plays a critical role in ASD (De Rubeis et al., 2014; Gaugler et al., 2014; Iossifov et al., 2014; Krumm et al., 2015; Grove et al., 2019). In the SFARI collection (https://gene.sfari.org/database/gene-scoring/), a total of 549 genes (from syndromic and category 1 and 2 groups) have been evidenced as strongly linked to ASD. In addition to diverse genetic factors, various environmental factors also contribute to ASD (Karimi et al., 2017; Modabbernia et al., 2017), rendering study of ASD even more complex and difficult. When investigating ASD etiology and therapeutics, age-dependent effects and homeostasis of neural activity also need to be considered. In this special Research Topic on “Autism Signaling Pathways,” 10 significant publications have been collected to summarize our current understanding of various aspects of ASD and to report on some new findings.

## Signaling Pathways and Neural Circuits in ASD

Filice et al. and Janickova and Schwaller have contributed articles related to the roles of parvalbumin and parvalbumin-positive interneurons (PV neurons in short) in ASD. Many autism mouse models display an altered number of PV neurons and/or expression levels of parvalbumin, suggesting involvement of PV neurons in ASD. The physiological significance of PV neurons to ASD is summarized by Filice et al. However, genetic evidence is still lacking to show whether mutation of the paravalbumin gene contributes to ASD. The study by Janickova and Schwaller showed that deletion of the parvalbumin gene results in increased oxidative stress in the brain of mice aged 3 or 6 months but not in younger mice, even though PV^−/−^ mice aged 1 month display autism-like behaviors. Thus, even though PV neurons are critical players in ASD, the disruption of PV neurons in ASD is likely caused by indirect neural circuit alterations as the nervous system tends to adapt to those changes to maintain homeostasis (Fazel Darbandi et al., 2018; Antoine et al., 2019).

As highlighted by Gandhi and Lee, the brain's cerebral cortex, striatum, thalamus, hypothalamus, amygdala, olfactory tubercle, ventral tegmental area (VTA), substantia nigra pars complex (SN), and cerebellum are all involved in ASD, though brain regions respond differentially to diverse genetic variations. Details of the signaling pathways, neural circuits, and anatomical changes related to repetitive behaviors in mice have been explored by Gandhi and Lee. They have also summarized the pharmacological treatments used for repetitive behaviors, representing potential therapeutic treatments for ASD. The corticostriatal circuitry was the focus of articles contributed by Mizuno et al., Brandenburg et al., and Caubit et al. Mizuno et al. investigated the transcriptomic profiles of BTBRTF/ArtRbrc mice as an ASD model, reinforcing the notion of complex signaling pathways and interconnectivity among many ASD-associated genes in the brain cortex and striatum. Brandenburg et al. utilized postmortem brains of ASD patients to analyze expression of GABA and serotonin receptors in the basal ganglia and their results indicate that alterations in an indirect pathway of the corticostriatal circuitry are involved in ASD. Finally, Caubit et al. applied the *Camk2a-Cre* transgene to selectively control expression of the zinc finger transcriptional factor TSHZ3, a high-confidence causative gene of ASD, providing a model to further characterize the impact of deleting *Tshz3* from specific cell types in terms of ASD phenotypes.

This special Research Topic also covers other ASD-associated molecules, including cell adhesion molecules (Gandawijaya et al.), G protein-coupled receptors (GPCR, DelaCuesta-Barrutia et al.) and NMDAR GluN2B (Bahry et al.). Gandawijaya et al. report how deletion of human chromosome 3p that results in absence of three closely related genes encoding neuronal immunoglobulin cell adhesion molecules, i.e. *Close Homolog of L1* (*CHL1*), *Contactin-6* (*CNTN6*), and *Contactin-4* (*CNTN4*), is relevant to ASD. DelaCuesta-Barrutia et al. summarize heterooligomer formation of various neurotransmitter receptors, such as the GPCRs of glutamate, dopamine, oxytocin and serotonin, and they discuss current advances in pharmacological approaches targeting GPCR heteromers. The impact of an early termination mutation at amino acid residue 724 of GluN2B on dendritic outgrowth is reported by Bahry et al. As a voltage-gated glutamate receptor, the finding that mutation of *GluN2B* promotes dendritic pruning is intriguing, but the underlying mechanism remains unclear and warrants further investigation.

Finally, Béroule discusses the paradoxical effects of the inflammatory cytokine IL-17A and anticonvulsant valproic acid (VPA) on the brain. Exposure to either IL-17A or VPA during pregnancy increases the likelihood of the offspring developing ASD, yet their effects on adult brains differ. Béroule has hypothesized that as a shared downstream target of IL-17A and VPA, type A monoamine oxidase (MAOA) exerts context-dependent paradoxical effects. He also discusses the roles of neuroinflammation and regulated MAOA expression in ASD.

## Perspective

**Figure 1:**
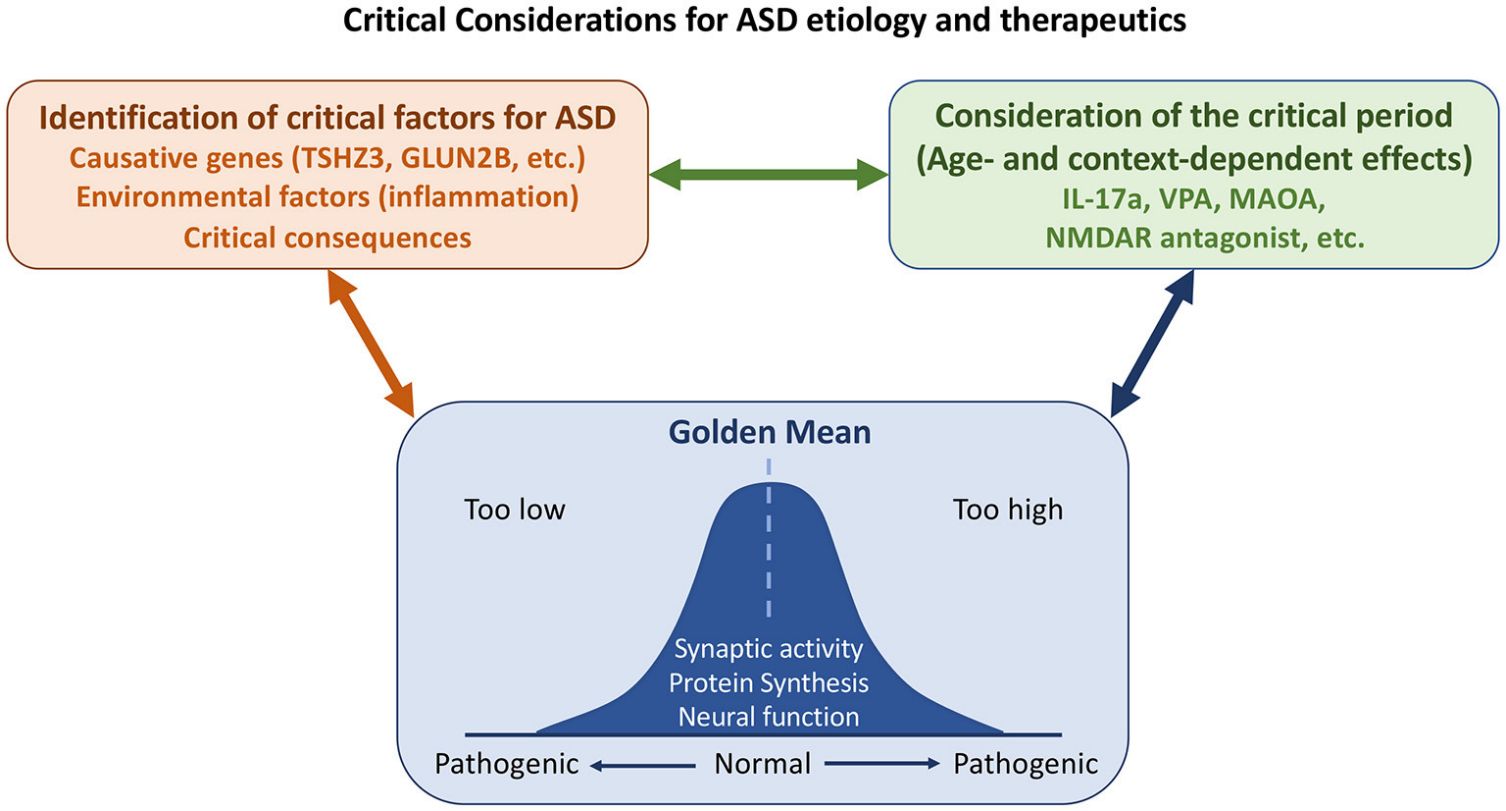
Some considerations on etiology, critical period, and homeostasis (the “golden mean”) for ASD study. Both genetic and environmental factors contribute to ASD. Some changes observed in patients and/or mouse models are consequences and not causes of ASD. It is essential to distinguish the causes of ASD from its consequences. Some causative genes and environmental factors that had been discussed in this Research Topic are indicated. As critical factors of ASD may act opposingly at different developmental stages or in different contexts, the critical period needs to be considered for ASD etiology and treatment. Finally, homeostasis of neuronal responses and nervous system activity must be maintained. Defects in either direction give rise to ASD pathological conditions.

Overall, ASD represents a highly complex group of disorders. Emerging studies in this Research Topic dissect their pathologies in neurotransmission (including both ion channels and GPCR), cell adhesion, transcriptional regulation, interconnections among different types of neurons and different brain regions. These studies provide foundations for future investigations. Certainly, other detailed discussions, such as chromatin remodeling and synaptic organization, development and signaling are not covered. Here, we further extend and emphasize three worth considering points for ASD research. First, what is the true cause of ASD? One should be cautious in interpreting results from postmortem brain tissues. Differences in postmortem tissues may be a consequence but not the cause of ASD pathology. Similarly, the aforementioned articles on PV neurons imply that altered parvalbumin-mediated regulation of oxidative stress does not cause ASD, even though PV neurons are indeed affected by ASD and are a feature of ASD phenotypes. Second, context- or age-dependent effects of ASD must be considered. Dr. Eunjoon Kim (Korea Advanced Institute of Science and Technology) and colleagues have shown that Shank2-knockout neurons exhibit NMDAR hyperfunction before weaning but NMDAR hypoactivity after weaning (Chung et al., 2019). Consequently, treatment with the NMDAR antagonist memantine exerted the opposite effects on Shank2-knockout mice before and after weaning. The differential effects of IL-17A and VPA on mice of different ages described by Béroule further echo this concept. Thus, to evaluate the effect of pharmacological treatment and signaling pathways of autism, age is one of critical factors for consideration. Finally, although it was not clearly addressed in the articles collected for this Research Topic, it is generally accepted that deviations from homeostasis (the “golden mean,” see Figure 1) are common in ASD. For instance, both increases and decreases in protein synthesis and signaling have been linked to ASD-associated neuronal defects (Lu and Hsueh, 2021). Synaptic morphology and responses must also be controlled within appropriate thresholds so that neuronal activity or function is neither excessive nor inadequate, since any such defects may lead to ASD pathological conditions.

## Author Contributions

Y-PH edited manuscripts published in the Research Topic, wrote the current manuscript, prepared the figure, and approved submission to the journal. Y-CL edited manuscripts published in the Research Topic, contributed discussion, and approved submission to the journal. All authors contributed to the article and approved the submitted version.

## Funding

This work was supported by Academia Sinica (AS-IA-106-L04 and AS-TP-110-L10) and the Ministry of Science and Technology, Taiwan (MOST 108-2311-B-001-008-MY3). Hussman Foundation grant HIAS18004.

## Conflict of Interest

The authors declare that the research was conducted in the absence of any commercial or financial relationships that could be construed as a potential conflict of interest.

## Publisher's Note

All claims expressed in this article are solely those of the authors and do not necessarily represent those of their affiliated organizations, or those of the publisher, the editors and the reviewers. Any product that may be evaluated in this article, or claim that may be made by its manufacturer, is not guaranteed or endorsed by the publisher.
